# The Jewish Hospital at Berlin

**Published:** 1912-02-24

**Authors:** 


### The Jewish Hospital at Berlin.

Of all denominational hospitals the world over, the best known is probably the old Jewish Hospital at Berlin, which through its surgeon-in-chief, Dr. Israels, has obtained a wide reputation and has become the Mecca of post-graduate students who are interested in kidney diseases and genito-urinary surgery. The building, however, is old and by no means ideal. In fact, considered from a modern hospital point of view, we know of few first-class hospitals that have to contend against so many structural defects. The wards are badly built and in consequence badly ventilated, and the site of the hospital is one of the worst possible. It has therefore been decided to build a new hospital. The Jewish community at Berlin is rich and has every justification for establishing a new hospital, since no special Jewish wards or kosher kitchens have been arranged for in the municipal hospitals as is the case in London, where at least three hospitals have made special provision for Jewish patients. The new building is placed at the corner of Exercierstrasse and Schulstrasse, and will accommodate nearly 300 patients. A feature of the plan is the fine mortuary block, which provides for adequate post-mortem and laboratory work, and contains several interesting new features of which we hope to give further details later on. Owing to the fact that the hospital is one of the recognised centres of post-graduate study in Berlin, accommodation has been provided for students in the shape of a large lecture theatre, and several working rooms. The administrative block, nurses' home, and isolation block plans are interesting because of the difficulties which have had to be overcome in view of the fact that the site is rather cramped. The provisional estimate for the building is £230,000. If the plans are rigidly followed, this institution ought to be one of the most interesting new hospitals on the Continent.

